# *LDB2* locus disruption on 4p16.1 as a risk factor for schizophrenia and bipolar disorder

**DOI:** 10.1038/s41439-020-00117-7

**Published:** 2020-09-29

**Authors:** Yasue Horiuchi, Tomoe Ichikawa, Tetsuo Ohnishi, Yoshimi Iwayama, Kazuya Toriumi, Mitsuhiro Miyashita, Izumi Nohara, Nanako Obata, Tomoko Toyota, Takeo Yoshikawa, Masanari Itokawa, Makoto Arai

**Affiliations:** 1 Schizophrenia Research Project, Tokyo Metropolitan Institute of Medical Science, Tokyo, Japan; 2 Department of Infection Control Science, Meiji Pharmaceutical University, Tokyo, Japan; 3 Laboratory for Molecular Psychiatry, RIKEN Brain Science Institute, Wako, Saitama Japan

**Keywords:** Schizophrenia, Schizophrenia, Bipolar disorder, Bipolar disorder

## Abstract

We had previously reported the case of a male patient with schizophrenia, having de-novo balanced translocation. Here, we determined the exact breakpoints in chromosomes 4 and 13. The breakpoint within chromosome 4 was mapped to a region 32.6 kbp upstream of the LDB2 gene encoding Lim domain binding 2. Variant screening in *LDB2* revealed a rare novel missense variant in patients with psychiatric disorder.

Schizophrenia is a chronic and disabling brain disorder that affects approximately 1% of the population. Although the disease mechanism is still unknown, its genetic predisposition is clearly evidenced. Genome-wide association studies have identified over 100 independent loci defined by common single-nucleotide variants (SNVs)^1^. A number of rare variants have been identified till date, with far larger effects on individual risk; de-novo mutations have also been reported to confer substantial individual risk^2,3^. Increasing evidence has suggested an overlap of genetic susceptibility between schizophrenia and bipolar disorder^4,5^. Most notable association has been found with the Disrupted In Schizophrenia 1 (*DISC1*) gene, based upon chromosomal abnormality with a balanced chromosomal translocation (1;11)(q42;q14.3) in a large pedigree^6,7^.

We had previously reported a male patient with schizophrenia, carrying a *de novo* balanced translocation t(4;13)(p16.1; q21.31)^8^. However, the exact breakpoint had not been determined till date. Here, we report the exact breakpoints on chromosomes 4 and 13 using next-generation DNA-sequencing analysis, in combination with fluorescence in situ hybridization (FISH) experiments on the patient. The estimated breakpoints were confirmed by FISH using the BAC clone (RP11-141E13), which was selected from the UCSC genome browser (GRCh38/hg38) (Fig. 1b). According to the database, the breakpoint on chromosome (chr) 13 was within the so-called ‘gene desert’ interval, where no known gene has yet been mapped. The breakpoint on chr 4 was mapped to the upstream region of a gene encoding a putative transcription regulator lacking a DNA-binding domain, namely *LDB2* (LIM domain-binding 2, also known as *CLIM1*) (Supplemental Fig. 1).

**Fig. 1:**
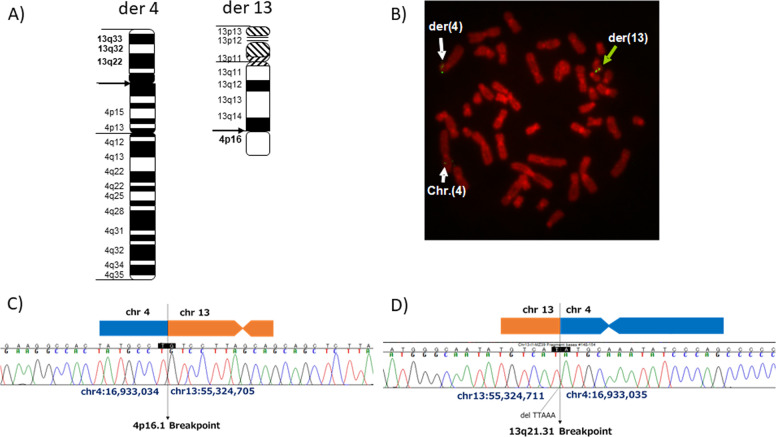
Determination of the chromosomal breakpoint in the patient with schizophrenia. **a** Idiogram of the translocation karyotype. **b** FISH analysis, using the lymphoblastoid cell lines (LCL) from the proband. Metaphase spread of LCLs showing BAC (RP11-141E13) hybridization signal to chromosome 4, derivative 4, and derivative 13. Loss of green signal (a white arrow) indicated deletion of this region. **c**, **d** Determination of breakpoint sequences of der (4) and der (13).

To determine the exact breakpoint, we conducted whole-genome sequencing using peripheral blood-derived DNA from the proband and the HiSeq 2500 (Illumina, CA, USA) as per the manufacturer’s recommended protocol. Confirmation of the translocation breakpoint and flanking sequence, and resequencing of *LDB2* were conducted by Sanger sequencing. Primer details are listed in Supplementary Table S2.

Fine mapping of breakpoints on chr 4 revealed chr 4:16,933,034 and chr13:55,324,705, which was 32.6 kbp upstream of *LDB2* (Fig. 1c). The breakpoint on chr 13 was located at chr 4:16,933,035 and chr13:55,324,711 (Fig. 1d). There was no nucleotide deletion or duplication at the breakpoint of chr 4. However, 5 base pairs (ttaaa) were lost from the chr13 breakpoint.

We further performed resequencing of *LDB2* to detect a rare variant (MAF < 0.005) having a major alteration in function of the gene. The subjects comprised of 520 unrelated Japanese patients with schizophrenia (SZ) (281 males and 239 females; mean age ± SD, 49.95 ± 12.00 years and 52.54 ± 13.29 years) and 423 with bipolar disorder (BP) (210 males and 213 females; mean age ± SD, 51.19 ± 13.36 years and 49.61 ± 13.94 years), diagnosed according to the Diagnostic and Statistical Manual of Mental Disorders, Fourth Edition (DSM-IV) with consensus from at least 2 experienced psychiatrists. Four hundred control subjects (183 males and 217 females; mean age ± SD, 40.48 ± 12.18 years and 40.63 ± 12.64 years) were included, whose second-degree relatives were free of psychosis, as reported by the subjects. All the participants provided written informed consent. This study was approved by the Ethics Committees of the Tokyo Metropolitan Institute of Medical Science and RIKEN. By resequencing of the coding region of *LDB2*, six rare variants (four synonymous and two nonsynonymous) were detected (Table 1). Of these, two variants (p.Thr83Asn and p.Ala287Ala) have not been reported in any database, including dbSNP (https://www.ncbi.nlm.nih.gov/snp/), gnomAD (https://gnomad.broadinstitute.org/), and jMorp (https://jmorp.megabank.tohoku.ac.jp/202001/variants) (Table 1). The two nonsynonymous variants (p.Thr83Asn and p.Pro170Leu) that were only found in BP were classified as probably damaging by PolyPhen-2^9^.

**Table 1:** Detected rare variant in *LDB2*.

Variant No.	Position in *LDB2*	Position of variant (GRCh37)	Coding DNA change	Amino acid change	dbSNP ID	MAF (in this study)			MAF (public database)	
						SZ	BP	C	gnomAD v2.1.1	jMorp
1	Exon 2	16760830	c.186C>T	p.Asp62Asp	rs144018108	0.001	0.0012	0	0.00100	0.00100
2	Exon 3	16597486	c.248C>A	p.Thr83Asn	NA	0	0.0012	0	NA	NA
3	Exon 3	16597383	c.351G>A	p.Thr117Thr	rs1287331315	0	0	0.0013	NA	NA
4	Exon 4	16590408	c.456G>C	p.Leu152Leu	rs771249608	0.001	0	0	0.00004	0.00040
5	Exon 4	16590355	c.509C>T	p.Pro170Leu	rs138524887	0	0.00120	0	0.00004	NA
6	Exon 7	16508563	c.861T>C	p.Ala287Ala	NA	0	0.00240	0	NA	NA

*MAF* minor allele frequency, *SZ* schizophrenia, *BP* bipolar disorder, *C* control, *NA* not applicable.

Previous reports had suggested 4p16.1 region to be associated with schizophrenia and bipolar disorder^10–12^; however, there was no association between SZ, BP, and control, in this study (Supplemental Table 1). Recent studies indicated that clinical significance of balanced chromosomal abnormalities was due to disruption of the topologically associated domains (TADs)^13^. Chromosomal breakpoint was located on the same TAD region as *LDB2*^14^, (Ohnishi et al. submitted), hence implying alteration of the gene expression of *LDB2*. Unfortunately, we could not collect RNA sample from the proband, due to which, we could not confirm the expression level of *LBD2*. Information regarding the function of LDB2 protein is limited, and several reports have shown LIM-domain proteins to regulate cell proliferation and cell fate in many regions of the CNS^15,16^. In support of our observation, study on the *Ldb2* KO mouse had suggested *Ldb2* deficiency to result in various behavioral and functional impairments relevant to mental disorders (Ohnishi et al. submitted).

In conclusion, we identified the breakpoint of balanced translocation t(4;13)(p16.1; q21.31), and proposed the *LDB2* gene to possibly be linked to psychiatric disorder; however, the correlation between phenotype and genotype regarding this disorder would require further studies.

## Supplementary information

Figure S1 Schematic representation of the translocation breakpoint loci on chromosome 4 (A) and chromosome 13 (B)

Table S1 Genotypic and allelic distributions of the <i>LDB2 </i>gene polymorphisms in the screening population

Table S2 Primer infomation for variant screening of <i>LDB2</i>

## Data Availability

The relevant data from this Data Report are hosted at the Human Genome Variation Database at 10.6084/m9.figshare.hgv.2909; 10.6084/m9.figshare.hgv.2912.
