# Supplementary figures and images for: LDB2 locus disruption on 4p16.1 as a risk factor for schizophrenia and bipolar disorder
Source: Hum Genome Var. 2020 Sep 29;7:31. doi: 10.1038/s41439-020-00117-7 (PMC7524746; doi:10.1038/s41439-020-00117-7)

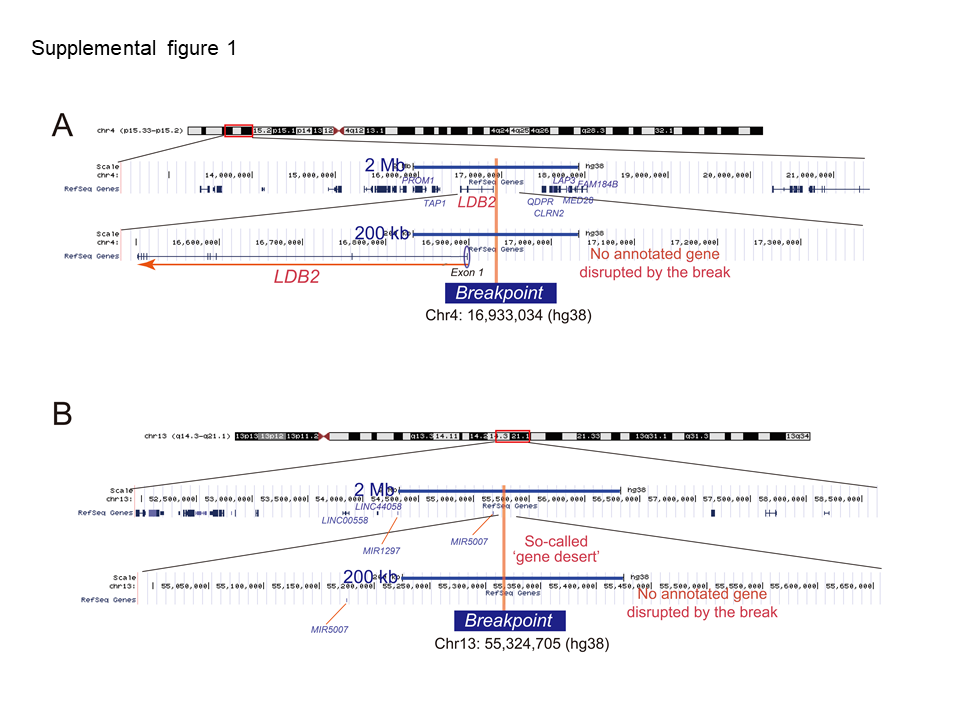

Supplement: Supplementary file 1 — Figure S1 Schematic representation of the translocation breakpoint loci on chromosome 4 (A) and chromosome 13 (B) [file 41439_2020_117_MOESM1_ESM.tif]
